# A qualitative exploration of the enablers of and barriers to conformance with antibiotic withdrawal periods on smallholding, peri-urban pig farms in Kiambu County, Kenya

**DOI:** 10.1371/journal.pone.0312362

**Published:** 2025-01-10

**Authors:** Claire Scott, Nicholas Bor, Kristen K. Reyher, Alex J. Tasker, Henry Buller, Irene Bueno, Lian F. Thomas

**Affiliations:** 1 The Bristol Veterinary School, University of Bristol, Langford, North Somerset, United Kingdom; 2 Animal and Human Health Department, International Livestock Research Institute, Nairobi, Kenya; 3 Department of Geography, University of Exeter, Exeter, United Kingdom; 4 Institute of Infection, Veterinary & Ecological Sciences, University of Liverpool, Leahurst Campus, Neston, United Kingdom; 5 Royal (Dick) School of Veterinary Studies, University of Edinburgh, Edinburgh, United Kingdom; UGHE: University of Global Health Equity, RWANDA

## Abstract

Non-conformance with antibiotic withdrawal period guidelines represents a food safety concern, with potential for antibiotic toxicities and allergic reactions as well as selecting for antibiotic resistance. In the Kenyan domestic pig market, conformance with antibiotic withdrawal periods is not a requirement of government legislation and evidence suggests that antibiotic residues may frequently be above recommended limits. In this study, we sought to explore enablers of and barriers to conformance with antibiotic withdrawal periods for pig farms supplying a local independent abattoir in peri-urban Nairobi. We drew upon semi-structured interviews with farmers and government animal health professionals as well as focus groups which involved private animal health professionals. We also explored farmers’ engagement with antibiotic withdrawal periods by visiting thirteen pig farms (supplying one of two local independent abattoirs) weekly for one month in order to capture instances of antibiotic use. We analysed data using reflexive thematic analysis. All farmers participating in the study demonstrated an awareness of the concept of antibiotic withdrawal periods and described intentions to conform, motivated by caring for others, wanting to prevent harm or a perception that regulation around antibiotic withdrawal periods existed for local independent abattoirs. The antibiotic use practices that we identified showed limited opportunities for non-conformance with antibiotic withdrawal periods. Farmers and veterinarians reported that instances of antibiotic use were uncommon, especially in slaughter-weight pigs, and were mainly restricted to the treatment of clinical signs under the supervision of an animal health professional. Local factors presented barriers to antibiotic withdrawal period conformance including farmers’ economic constraints, lack of formal medicine recording, an absence of consistent abattoir monitoring and resource emergency, such as water scarcity on farms. This study demonstrates the importance of these contextual factors to conformance with antibiotic withdrawal periods. We highlight the need to account for farm-level influences when planning future research and interventions aimed at reducing the presence of antibiotic residues in meat from smallholding pig farms in peri-urban Nairobi.

## Introduction

Antibiotic withdrawal periods (ABWPs) are defined as the time between the final administration of an antibiotic to the point at which the animal can be slaughtered for human consumption so that antibiotic residues in the carcass are within safe limits for the consumer [1]. If consumed, antibiotic residues have the potential to cause toxicities, allergic reactions, disruption of gut microflora and the development of antibiotic resistance which could lead to antibiotic-resistant infections [2]. Whether cooking destroys antibiotic residues is believed to vary by antibiotic compound and cooking technique [3, 4]. ABWPs are derived from the pharmacokinetic properties of each antibiotic as well as maximum residue limits for each antibiotic in animal tissue [5, 6]. Conformity to ABWPs represents a globally recognised infrastructure to protect consumer safety.

Kenya does not currently have government legislation stipulating conformance with ABWPs for pigs being sold to the domestic market; legislation only covers those intended for export [7]. Therefore, conformance with ABWPs for pigs being slaughtered for the domestic market should be considered a non-binding, best-practice guideline. As such, Kenya’s ‘Guidelines for the prudent use of antimicrobials in animals’ calls for the elimination of antibiotic residues using ABWPs [8]. ABWPs are generally found displayed on antibiotic packaging (or insert slips), however, the communication of ABWPs is made more complex by findings that ABWPs are variably displayed on medicines in Kenya [9, 10].

In Kenya, two groups of animal health professionals (AHPs) exist. The first are veterinarians who are legally permitted to prescribe and administer medicines to pigs [11]. In contrast, veterinary para-professionals–commonly known as animal health assistants or ‘para-vets’–are only legally permitted to administer injections and medicines to pigs “under the responsibility or direction” of a veterinarian [11, p. 28]. Non-AHPs are not legally permitted to administer medicines to pigs [11]. However, studies in Kenya have reported that veterinary para-professionals prescribe medicines to farms without supervision by a veterinarian [12–14] and that farmers commonly purchase and administer antibiotics to farm animals [9, 10].

To supply the domestic market in Nairobi, Kenya, local independent abattoirs (LIAs) near to the city source pigs mainly from small-scale farms in their surrounding neighbourhoods [15–17]. LIAs are only regulated by local governmental legislation, meaning that conformance with ABWPs is not legally mandated [7]. Murungi et al. [16] and Sentamu et al. [17] have detailed how LIAs lacked infrastructure which could lead to negative impacts on food safety. Reflecting this, equipment and personnel required to monitor antibiotic residues were not present in the LIA studied by Bor et al. [18] at the time of their study. LIAs have been characterised as using informal pig sourcing where farmers can sell pigs to brokers who, in turn, sell pigs to traders [16]. Traders often submit pigs from several farms to the abattoir, pay for the animals’ slaughter and then sell the meat onto selling outlets, such as butchers or restaurants. When combined with a lack of farm-specific animal identification, this results in significant challenges for farm-to-processing traceability [19].

Conversely, one large integrated processor operating in the county rears its own pigs, rears pigs through contractors and buys pigs directly from farms [16, 20], meaning that, for them, farm-to-processing traceability is possible. This large integrated processor supplies both Kenyan pig export markets and high-end pork meat to supermarkets [16], therefore requiring compliance with export meat laws on conformance with ABWPs [7]. An example of such a control includes the issuing of ‘no objection’ permits from government AHPs before travel to slaughter [16] which certify, amongst other regulations, that recent antibiotic use (ABU) has not taken place.

Recent studies have raised concerns around conformity to ABWPs for pigs slaughtered in LIAs around Nairobi [16, 18, 21]. Interviewed brokers have reported that ABWPs were often not observed by farmers selling pigs to a LIA [16]. A pilot study by Irungu et al. [21] found that 24/48 beef, poultry and pig farmers in counties around Nairobi reported they sell animals for meat immediately after treatment. Finally, Bor et al. [18] tested pork meat juices from a LIA and found 41% of samples (adjusted for diagnostic test performance) tested positive for antibiotic residues above EU legal limits. As a comparison, studies over the last 20 years investigating the prevalence of antibiotic residues from milk from smallholding dairy farms in Kenya identified residues in 5–16% of samples [22–26]. Predictions that pork production in Kenya will increase dramatically [20, 27] coupled with the findings of Bor et al. [18] around antibiotic residues suggests the need for further research. To provide further insight into this specific food safety concern, we aimed to explore factors shaping conformance with ABWPs for pigs slaughtered at the LIA studied by Bor et al. [18].

By exploring these factors in a qualitative scoping study, we aimed to gain a contextualised understanding of the enablers of and barriers to conformance with ABWPs experienced by both farmers and AHPs. This, in turn, could provide a foundation on which to base future research to derive evidence-based recommendations and interventions aimed at improving conformance with ABWPs beyond current levels.

## Methods

The description of methods which follows can also be found in our related manuscript [28].

### Farm visits

In their examination of this specific pork value chain, Murungi et al. [16] found that 80% of the pigs slaughtered in the abattoir studied by Bor et al. [18] were sourced from farms in Kiambu County, a peri-urban area of Nairobi. We, therefore, chose to base our study in this county. We recruited farms into the study which supplied pigs to a LIA and were based in one of the four sub-counties of Kiambu County closest geographically to the LIA studied by Bor et al. [18]. These were Kabete, Kikuyu, Kiambaa and Limuru. Given that a further LIA had begun operating in the county since the study by Bor et al. [18], we also chose to accept participants who supplied pigs to this LIA.

Government AHPs drew on their knowledge of pig farms in the local area to assist CS and NB to recruit farmers into the study. Government AHPs were asked to approach any farmers who kept pigs in one of the four sub-counties described above and supplied pigs to a LIA. Alongside government AHPs, CS and NB visited those farmers who had expressed interest in the study and asked them for their participation through written informed consent after reading aloud a letter informing potential participants of the study in English, Swahili or Kikuyu (a local tribal language). Due to resource and time constraints to complete this scoping study, recruitment was stopped at the end of our allocated time for participant recruitment. This resulted in recruitment of 13 farms into the study. Nine out of 13 interviews were carried out in English (the first language of the lead researcher—CS). Where required, Swahili translation was provided by a research assistant with knowledge of the project aims and objectives (NB). At the initial interview, we explored characteristics of farm management with farmers. During this initial visit, we also explained our intention to capture all instances of medicine use completed on the farm for the next month. Capturing medicine (specifically antibiotic) use practices enabled us to triangulate the general discussions elicited during farmer and AHP interviews with farmers’ real-life, contextualised engagement with ABWPs. Each reported instance of ABU was qualitatively explored in order to understand ABU practices, particularly whether pigs may have been sent to slaughter within an ABWP. No single method to determine ABU at the farm level has been identified as most effective for this context, therefore we trialled multiple methods (waste bucket analysis, medicine recording, weekly semi-structured interview and the ‘Drug Bag’ medicine sorting technique) in parallel. Further description and evaluation of these methods can be found in our related manuscript [28]. At the initial visit, we left waste buckets at the recruited farms for the farmers to place empty medicine packaging, as well as medicine-recording sheets to record instances of medicine use. The medicine-recording sheet and a photo of the waste bucket, clipboard and associated signage can be found in Supporting Information (S2 File).

CS and NB visited farms a further four times (approximately weekly) over the following 28–31 days to understand pig health care practices relating, in particular, to ABU and ABWP conformance. Government AHPs did not accompany us for further visits to farms. During the weekly farm visits, we conducted semi-structured interviews to explore events over the previous week, including instances of medicine use and selling or slaughter of pigs. We also held discussions with participants about any medicines that had been placed in the waste bucket or recorded on the medicine-recording sheets. By visiting farms weekly, we aimed to minimise the possibility that participants would forget events such as instances of ABU or selling of pigs.

On the final visit, in addition to repeating the research activities which we carried out at the weekly visits, CS and NB carried out the ‘Drug Bag’ medicine-sorting technique [29] with participants. To do this, CS and NB visited every agrovet store in the study area (n = 15) which they could locate by searching Google Maps [30], using NB’s local knowledge of the study context and asking participants which stores they used. Antibiotics were purchased (n = 36) or photographed (n = 6) and were numerically labelled. Participating farmers were asked to sort through the contents of the bag (as per Dixon et al. [29]) and categorise medicines they recognised and those they didn’t recognise. Then, participants were asked to sort the medicines from the pile they recognised into those they had used for pigs and those they had not used for pigs. Participants then sorted the pile of medicines they had used for pigs into those they had used frequently and those they had not used frequently. The final sort was then recombined into the pile of antibiotics participants had used for pigs and participants were asked to sort this pile again, but this time into the medicines they had used in the last month and those not used in the last month for pigs. Qualitative discussion around ABU practices, especially to understand ABWP conformance, was held throughout the sorting process.

In order to gather reliable and robust data, qualitative research often benefits from the establishment of trust between participants and researchers. We aimed to build trust between participants and researchers through our weekly visits, in order to facilitate frank and open discussion. This was especially relevant for our discussions around farmer awareness, intentions and experiences around ABWP conformance, which we held as the final activity on our last visit to farms. This was carried out on our final visit to ensure that our questions about ABWP conformance did not affect farmers’ ABU practices and engagement with ABWPs over the previous study period.

Interview schedules can be found in Supporting Information (S1 File). Interviews were audio recorded and field notes were also written by CS in the form of a reflexive diary. Farm interviews lasted between ten and 50 minutes and took place shortly before and during the ‘short rains’ of October to November 2022. As time compensation for farmers completing the study, we gave farmers protective boots, a scrubbing brush and disinfectant which totalled approximately 12 USD in value upon completion of the final visit. We also created a feedback booklet based on our observations throughout the project which we gave to participants at the end of the final visit. This can be found in Supporting Information (S1 Fig).

### Focus group discussions with animal health professionals

We included FGDs with AHPs in order to explore current roles and possible alternative roles of AHPs relating to conformance with ABWPs; understand the context of on-farm findings within the wider system; and triangulate data between farmers and AHPs. CS and NB completed two FGDs with AHPs working with pigs in the county, one was with private veterinarians and the other was with private para-veterinary professionals. Participants were recruited by: visiting agrovet stores in Kiambu County to ask AHPs working in the store for their participation; through snowball sampling; by posting into social media groups for AHPs in the county; and through our own contacts. Participants included AHPs of both genders and those who primarily worked with farmers in the field as well as those who worked primarily from agrovet stores. Recruitment of participants was stopped at the end of the allocated time for recruitment of participants for FGD, which resulted in us recruiting seven private veterinarians and five private para-veterinary professionals. As compensation for their time, veterinary para-professionals were given KES 1000 (approximately 8 USD) and private veterinarians were given KES 2000 to attend the FGD which lasted approximately two hours and took place at the International Livestock Research Institute. FGDs were carried out in English and were audio recorded. Field notes (in the form of a reflexive diary) were also taken by CS. The FGD guide can be found in Supporting Information (S1 File).

### Key informant interviews with animal health professionals

We included key informant interviews (KIIs) with government AHPs to explore their role, opinions and experiences around conformance with ABWPs in this context. We completed three semi-structured KIIs with the government veterinarians responsible for each of the four sub-counties we visited during the study (one government veterinarian supervised two sub-counties). We recruited participants for KII by visiting government veterinarians at their place of work. Participants for KII were each given KES 1000 as time compensation. KIIs lasted between 35 minutes and one hour and took place at the government veterinarian’s place of work. KIIs were carried out in English and were audio recorded. Field notes were also taken by CS in the form of a reflexive diary. KII guides can be found in Supporting Information (S1 File).

### Data management and analysis

CS transcribed audio recordings from farmer interviews, FGDs and KIIs with the assistance of a digital recording software [31]. Interviews were transcribed during the same week that they were completed, to ensure an iterative process between data collection and data analysis. CS analysed data qualitatively, by inputting transcripts and field notes into NVivo qualitative analysis software for thematic analysis [32]. After a process of data familiarisation, CS coded data using Braun and Clarke’s version of reflexive thematic analysis [33, 34], with input from the research team to consider alternative layers of meaning. Data were considered from a critical realist stance–that realities exist but culture, language and human practices shape how we experience, perceive and contextualise ‘truths’ [33]. Data were coded inductively, according to the research question: ‘What are enablers of and barriers to conformance with ABWPs for pig farmers supplying a LIA in Kiambu County, Kenya?’ This resulted in 41 individual codes. From the codes generated, CS generated six themes which described patterns of shared meaning that she interpreted to underpin our data [34].

In order to contextualise qualitative data, CS also semi-quantitively examined each instance of reported ABU captured during our farm visits. Within Microsoft Excel [35], CS tabulated the reported ABU practices associated with each instance of ABU, including the length of the ABWP of the antibiotic and the characteristics of the pigs treated, in order to calculate whether pigs may have been sent to slaughter within an ABWP. CS also recorded details of pig sales to a LIA over the study period for each holding; each sale was assessed against reported instances of ABU for those pigs, to understand whether pigs may have been sent to slaughter within an ABWP. Finally, CS triangulated between these two data sets and considered where semi-quantitative data enriched qualitative findings.

### Ethics statement

This study gained ethical approval from the International Livestock Research Institution Institutional Research Ethics Committee (ILRI-IREC2022-16) and was accredited by the National Commission for Science, Technology and Innovation in Kenya (NACOSTI P/22/18768). Permission to complete the study was obtained from the relevant veterinary bodies in Kenya. Written informed consent was obtained for all participants. The recruitment period for the study was from 21^st^ September 2022 to 23^rd^ November 2022.

### Positionality statement

The research team was a collaboration between researchers from Kenya and the UK. NB and LT had extensive experience conducting research in the Kenyan setting and led the study appraising the prevalence of antibiotic residues at a LIA which served as rationale for the current work [18]. CS, KR, AT, IB and HB have experience completing qualitative research examining farm animal ABU. The team was comprised of six veterinarians by background (CS, NB, KR, AT, IB, LF), two of whom have social science expertise (CS, AT), and one social scientist (HB).

The lead researcher (CS) is a white, British, female veterinarian who was completing qualitative research at PhD level at the time of the study. She had experience completing thematic analysis from her UK-based PhD research examining farm animal ABU. CS had not visited the community being studied prior to completing this research, meaning that she can be considered an ‘outsider’ [36] to the Kenyan context. This allowed her to question taken-for-granted beliefs held by members of the community, for example realising that ABWPs were not legally mandated for pigs not intended for export in Kenya, which altered the lens through which she completed her enquiries. Given this ‘outsider’ positionality, CS was dependent on those within the community to provide contextual understanding throughout the study (especially during study planning) and to carefully translate interviews completed in Swahili. NB was particularly instrumental to this (NB accompanied CS throughout the project), as was LT and those acknowledged at the end of this work. Their input and guidance, as well as in-depth qualitative discussion with participants, allowed CS to carry out the research sensitively to the participants in the study and to consider the potential for lost meaning or misinterpretation alongside her iterative interpretation of the qualitative data.

### Inclusivity in global research

Additional information regarding the ethical, cultural, and scientific considerations specific to inclusivity in global research is included in the Supporting Information (S1 Checklist).

## Results

### Farmer and farm characteristics

Ten out of 13 farmers exclusively supplied one of two LIAs operating within Kiambu County. Three out of 13 farmers predominately supplied pigs to a large integrated processor operating within the county, although they also sold pigs to a LIA in specific circumstances such as to slaughter ex-breeding animals or if the larger integrated processor was fully booked. Participating farms housed two– 72 (median 23) pigs at the time of the study, which was characterised as small- to medium-scale enterprises during professional discussions with AHPs operating in the area. (Small farms were described as housing zero– 50 pigs, medium farms kept between 50 and 200 pigs and large farms housed more than 200 pigs.) The main interviewees for farms were made up of eight men and five women; other viewpoints were opportunistically captured from other farmers in attendance once informed consent was obtained. Nine out of the thirteen farmers owned or jointly owned the farm; the other four participants were employed to work on the farm. Table 1 shows data collected around farm characteristics (see also Scott et al., [28]). A map showing the farms visited during the study can be found in our related manuscript [28]. All anonymised and de-identified data have been made available in a data repository which can be found at: https://reshare.ukdataservice.ac.uk/857083/.

**Table 1:** Summary of themes describing enablers of and barriers to conformance with ABWPs.

Theme	Description
**Enablers of conformance with ABWPs**	
**Theme 1: Farmers are aware of the concept of ABWPs**	• All farmers and AHPs demonstrated awareness of the concept of an ABWP • Most (8/13) farmers could identify the ABWP from an item of antibiotic packaging • Farmers described how AHPs frequently made farmers aware of the ABWP when administering antibiotics to pigs
**Theme 2: Farmers want to conform with ABWPs**	• Farmers appeared strongly motivated to conform with ABWPs ○ For reasons of food safety ○ To follow perceived regulations and enforcement at the LIA
**Theme 3: ABU practices lead to ABWP conformance ‘by default’**	• ABU in pigs close to slaughter appeared uncommon ○ ABU was not broadly reported for growth promotion or prophylaxis ○ Disease in slaughter-weight pigs was described as uncommon ○ Disease sometimes remained untreated when it occurred
**Barriers to conformance with ABWPs**	
**Theme 4: Farmers may be unaware of specific ABWPs**	• Some farmers did not understand specific ABWP wording on packaging • AHPs may not make farmers aware of ABWPs • AHPs may not make farmers aware that they have administered an antibiotic • Antibiotics may be included in products without suitable labelling • ABWPs may not be reliably displayed on antibiotic packaging
**Theme 5: ABU practices challenge conformance with ABWPs**	• Lengthy ABWPs of commonly used antibiotics in the context of rare medicine recording and recall problems • Potential for higher ABU at specific risk periods not captured by research • Potential for the extent of ABU to be under-reported by participants in our study
**Theme 6: Economic fragility and resource constraints challenge ABWP conformance**	• Delaying slaughter may be too expensive amidst high production costs • Resource scarcity may lead to emergency sale of pigs still within ABWPs
**Theme 7: Poor regulatory frameworks may weaken farmer motivation to conform with ABWPs**	• Lack of enforcement at LIAs may weaken farmer or broker/trader motivation to conform • Enforcement at large integrated processor may lead to LIAs being used as salvage for treated, non-recovering pigs

Abbreviations:

ABU: Antibiotic use

ABWP: Antibiotic withdrawal period

AHP: Animal health professional

LIA: Local independent abattoir

### Enablers of and barriers to conformance with ABWPs

Our results are laid out in terms of the seven themes we developed from a process of thematic analysis. Three themes describe enablers of conformance with ABWPs and a further four themes describe barriers. Table 1 summarises these themes. Alongside the farm characteristics, Table 2 shows a summary of the instances of ABU and the number of pigs sold for each farm.

**Table 2:** Characteristics of farms and farmers recruited for the study alongside their awareness of antibiotic withdrawal periods.

Farm number	Farm sub-county	Participant role on the farm	Participant gender	Main outlet for pigs (LIA / LIP)	Number of pigs present on first visit				Number of pigs sold for slaughter during project		Total number of reported instances of ABU (see Scott et al., pre-print for further details)	Days between first and last visit	Language of interviews	Could the participant identify the ABWP from an item of antibiotic packaging?	Does the participant keep antibiotics on the farm to administer to pigs themselves?
					Total	Sows > parity 1	Unweaned piglets	Other (weaners/growers/ finishers/boars	To LIA	To LIP					
1	Kiambaa	Farm hand	Male	LIA	51	3	23	25	4	0	4	28	Swahili	No	No
2	Kiambaa	Farm hand	Female	LIA	18	1	0	17	0	0	0	28	Swahili	No	No
3	Kabete	Farm owner	Male	LIA	16	2	14	0	0	0	0	28	English	Yes	No
4	Kabete	Joint farm owner	Female	LIA	35	3	9	23	3	0	1	28	English	Yes	No
5	Kiambaa	Farm hand	Male	LIA	13	0	0	13	7	0	9	28	Swahili	No	No
6	Kabete	Farm owner	Male	LIP	74	0	0	74	0	20	2	28	English	Yes	Yes
7	Kabete	Farm owner	Male	LIP	50	2	12	38	0	12	4	28	English	Yes	Yes
8	Limuru	Farm owner	Female	LIP	40	1	9	30	0	20	2	31	English	Yes	Yes
9	Limuru	Farm manager	Male	LIA	15	0	0	15	0	0	1	31	English	Yes	Yes
10	Kikuyu	Farm owner	Female	LIA	23	1	13	9	0	0	2	28	Swahili	Yes	No
11	Kikuyu	Farm owner	Female	LIA	34	3	16	15	0	0	2	28	English	Yes	No
12	Kikuyu	Farm owner	Male	LIA	16	2	0	16	0	0	4	29	English	No	No
13	Kikuyu	Farm owner	Male	LIA	2	0	0	2	0	0	1	29	English	No	No
Totals					387	18	96	277	14	52	32				

Abbreviations:

SSI: semi-structured interview

LIA: local independent abattoir

LIP: large integrated processor

ABWP: antibiotic withdrawal period

#### Enablers of conformance with ABWPs

*Theme 1*: *Farmers are aware of the concept of ABWPs*. There are likely to be circumstances where farmer awareness of an ABWP is a necessary prerequisite for the farmer to conform with the ABWP, such as when a pig recovers from disease before the end of the ABWP of the antibiotic administered to treat it. Since approximately 60% of the Kenyan population have been reported to keep livestock [27], livestock farming is not considered a specialism in Kenya, but is part of daily life. Farmers selling to a LIA in our study were generally busy, navigating multiple employments, activities and responsibilities. Therefore, we did not expect farmers to engage in detailed discussion of ABWPs with us during the study, especially given the lack of governmental legislation in this area. Despite this, all farmers described to us the concept of an ABWP: that a delay is required between the administration of an antibiotic and slaughter. This was reflected by AHPs, who reported that most farmers were aware of ABWPs and that farmers buying antibiotics from an agrovet (veterinary medicines store) frequently asked them for the ABWP.

*“When you talk to farmers they also know the effects of using antibiotics in their animals and taking them immediately to slaughter.”* (KII, Government Veterinarian 1, November 2022)

The length of time of each ABWP is specific to the antibiotic and should be displayed on the packaging. In our study, we specifically did not ask farmers to define an ABWP but asked farmers to describe their actions following a need to treat a pig with a medicine close to slaughter. Therefore, farmers demonstrated an understanding of the concept of an ABWP without necessarily understanding the specific technical language, by answering that they would not send the animal immediately to slaughter and detailing why. Farmers could demonstrate further understanding of ABWPs by describing that the length of the ABWP varied, should be displayed on antibiotic packaging and by explaining the meaning of ABWP labelling when shown an item of antibiotic packaging with the antibiotic withdrawal period correctly displayed.

In our study, eight out of the 13 (62%) participating farmers described to us that the withdrawal period for each antibiotic varied and that the specific time for the ABWP should be detailed on the packaging. They also correctly explained the meaning of ABWP labelling on the antibiotic packaging that they were shown. Importantly, this included all of those participants who kept antibiotics on the farm and reported administering these to pigs themselves. These tended to be larger farms that mainly supplied pigs to the large integrated processor (see Table 1).

*“The vet mentioned it [the ABWP] to me and, because the vet knew I was treating my pigs, he had to educate me. […] I remember, once you administer, read the instructions.”* (Interview, Farmer 8, November 2022)

Providing insight into participants’ awareness of ABWPs, several farmers reported this to be as a result of discussions with AHPs, although it was frequently unclear whether farmers were referring to discussions held with a veterinarian or a veterinary para-professional.

*“So he [the AHP] tells me: this [pig] should go two weeks. This [pig], it must stay here one month. So that if he [the AHP] goes and someone comes to buy the meat, I tell them, ‘No. Just wait for the medicine for the pig to go*.’” (Interview, Farmer 13, November 2023)

As the quote demonstrates, participants described how AHPs commonly made them aware of the specific ABWP for each medicine at the time of prescribing and explained that these discussions and the advice given by AHPs influenced their practices.

*Theme 2*: *Farmers want to conform with ABWPs*. In cases when a pig recovers from disease before the end of the ABWP of the antibiotic administered to treat it, farmer intention to conform with ABWPs is also a necessary prerequisite for the ABWP to be conformed with. Despite ABWPs not being in law for the domestic market for pigs in Kenya, all farmers in the current study described an intention to conform with ABWPs. Most farmers cited that they were motivated to conform with ABWPs due to food safety concerns and caring for those around them. Farmers often described how these caring intentions were influenced by their religious faith and national values.

*“Because you are causing harm to another person knowingly. And us, you know we are Christians.”* (Interview, Farmer 9, November 2022)

Other farmers similarly reported intentions to conform with ABWPs, but described this to be due to concerns around repercussions from non-conformance, seemingly believing that a system of monitoring and enforcement at the LIA was present under perceived regulations:

*“Because once you slaughter, it will show. I know, the meat will show. […] The vet knows. They will tell you: ‘You have given medicine yesterday, or before 3 days’. They will tell you. I have gone to their slaughterhouse and I have seen the vet saying this meat cannot be consumed. The reason? They had administered some antibiotics before 3 days.”* (Interview, Farmer 8, November 2023)

Participating farmers’ intentions around ABWPs were reinforced to us by stories from three participants who described cases in which their intention to conform with ABWPs meant that they did not send pigs to slaughter at a LIA when they might have wished to.

*“There were three, they came ill. And when they came ill the vet come and he injected them. Now we saw that they are not improving. The time when we wanted to sell them, my husband told me, because these ones they have not even finished one month, even one week, before they were injected. Let them die and then we bury them.”* (Interview, Farmer 11, November 2022)

This was despite the precarious financial circumstances in which many of the farming participants in our study lived and the monetary value that fully grown pigs represented.

*Theme 3*: *ABU practices lead to ABWP conformance ‘by default’*. An understanding of when, how and why antibiotics are used is crucial to gaining an appreciation of factors shaping ABWP conformance because, for non-conformance with an ABWP, an antibiotic must have been administered and the pig slaughtered within the ABWP for the specific antibiotic. Broadly, participants in our study characterised ABU practices as demonstrating few opportunities for non-conformance with ABWPs at a LIA. This was due to reportedly infrequent use of antibiotics, especially for pigs close to slaughter. Both farmers and AHPs reported that ABU for pigs was avoided and minimised where possible. This was the case for several indications of ABU, including ABU for growth promotion, prophylaxis of disease and treatment of disease.

For ABU for growth promotion or for prophylaxis of disease in pigs, no farmers reported completing frequent (more than six-monthly), routine ABU. This was despite farmers being able to purchase antibiotics at agrovet stores without veterinary prescription (see also [13, 37]) and the availability of products containing antibiotics in the study area which were labelled with indications for use such as to increase growth and improve feed conversion (see also [37]). Farmers did practice measures to combat under-productivity, however, in our experience, these did not include intentional ABU.

*“I have not heard about use of antibiotics for growth in pigs […] but I know that they’re used when the pigs are sick. They have also, also, um, farmers have this notion that pigs don’t get sick… They don’t see the needs for antibiotics for pigs.”* (KII, Government Veterinarian 3, November 2022)

During our study, participants discussed farmers’ wishes to avoid ABU for growth promotion, prophylaxis or treatment of disease, due to wishing to avoid the costs associated with ABU. This was described in the context of notable financial pressure affecting pig farmers. Further, as the previous quote demonstrates, participants reported a view that infectious diseases in pigs were uncommon, meaning that antibiotics were rarely necessary for the treatment of disease. This was despite reports of notifiable disease in the area, our own observations of extremely emaciated pigs (see also [38]), extensive pig movement due to the need to transfer genetic material (see also [20]), poor biosecurity and very low reported rates of vaccination. Farmers described that pig health could broadly be maintained without antibiotics:

*“You keep them warm, you keep them clean, you feed them well. They are there to go. No medicine.”* (Interview, Farmer 3, November 2022)

The lack of need for medical treatment was expressed to be especially the case for pigs nearing slaughter weight; most infectious disease and instances of ABU were discussed by both farmers and AHPs as occurring in piglets. In these cases, surviving piglets were held on-farm until far beyond the ABWPs, or died and were reportedly disposed of. It is unlikely, therefore, that these instances of ABU represented non-conformance with ABWPs at a LIA. FGD participants described these instances of ABU in piglets not destined for slaughter anytime soon as ABWP conformance ‘*by default’* (FGD, Veterinary Para-professional, November 2022).

Also driven by economic difficulties, where clinical signs of disease did occur, farmers often did not seek AHP advice. Four out of 13 farmers kept a small selection of antibiotics on their farms and administered these in line with their previous antibiotic experiences. As previously discussed, these farmers all understood the meaning of ABWP guidelines on packaging and described intentions to conform with ABWPs. For other farmers, the financial barrier to seeking AHP assistance often led to clinical signs of disease being left untreated. One participant described how some pigs would live without antibiotic treatment and others would die with antibiotic treatment.

*“You have to pass through challenges. And the challenge also makes you remember God more. So when you are smooth sailing, you are just settled, God, thank you for the day.”* (Interview, Farmer 4, November 2022)

As the quote above demonstrates, these challenges were considered part of the tapestry of farming and life; ‘antibiotic non-use’ was just as interesting to explore as ABU (see also [39]). For example, occasionally, farmers cited goals of low medicine use–aimed at avoiding antibiotic resistance or antibiotic residues–as reasons to avoid ABU.

Participant: *“I hate just always giving the medicines*. *I hate that*. *Ok so you do that for a very long time*, *it becomes*, *what do you call it*? *Too much drugs*…*”*

Interviewer (CS): *“Resistance*?*”*

Participant: “*Yeah*, *yeah*. *It will resist*.*”* (Interview, Farmer 6, October 2022)

Cost is likely to be a notable driver of these goals as, when asked why the same farmer preferred to bring a singular pig into a passageway to treat it with oral antibiotics rather than treat the whole group of pigs, the farmer said:

*“Because if maybe you give all of them [the antibiotic], that’s an extra cost too. So I have to be specific to this one [pig].”* (Interview, Farmer 6, October 2022)

As the above quote demonstrates, farmers in our study also described the need to isolate antibiotic treatment to as few animals as possible for the shortest time, in order to lessen the financial burden of such interventions.

#### Barriers to conformance with ABWPs

*Theme 4*: *Farmers may be unaware of specific ABWPs*. We identified several areas as having the potential to challenge farmer awareness of the specific ABWP of antibiotics administered to pigs. These fell into four categories which were: farmers not demonstrating the ability to identify the specific ABWP from antibiotic packaging; AHPs not reliably informing farmers of ABWPs; mislabelling or concealment of the active ingredients of substances containing antibiotics used for pigs; and ABWPs not being reliably displayed on antibiotic packing.

For farmers not demonstrating an ability to identify the specific ABWP from antibiotic packaging, despite having previously demonstrated an understanding of the concept of an ABWP, five out of the 13 farmers participating in the study could not explain the meaning of an ABWP from its indication on antibiotic packaging. Rather than detailing that the length of the ABWP varied between antibiotics, these participants said a more arbitrary time period for the ABWP, which ranged between participants from three days to six months.

It is important to note that each of the five farmers who were unable to explain the meaning of an ABWP from its indication on antibiotic packaging reported that they always called an AHP to administer medicines, which was also confirmed by our observations. In such cases, it is the responsibility of the AHP to make the farmer aware of the ABWP for the specific antibiotics being administered. In our FGDs and KIIs, however, AHPs reported that whether or not AHPs consistently made farmers aware of the specific ABWP varied. In our study, one participant described that:

*“It depends on the individual integrity of the vet or the para-vet*.” (FGD, Private Veterinarian, November 2022)

Veterinary para-professionals were of the view that veterinarians were unlikely to explain to farmers about ABWP, saying veterinarians are:

*“…not that concerned about explaining to the farmers about withdrawal periods.”* (FGD, Veterinary Para-professional, November 2022)

In a separate FGD, veterinarians described their experience that veterinary para-professionals did not inform farmers about ABWPs:

*“According to my own experience, I have never heard any para-vet explain to the farmers what is withdrawal period. They just treat and go.”* (FGD, Private Veterinarian, November 2022)

AHPs in our study also reported that uncertified AHPs–whom they termed ‘quacks’–operated in the area as a result of poor regulatory enforcement (see [9, 14, 40, 41] for similar findings in Kenya and East Africa).

*“So they [the farmer] will sometimes even call a quack. Somebody who is not trained. We have a problem that the veterinary practice in Kenya is not very well regulated. So anybody starting, even somebody who has just gone to college and, uh, they have learned animal production, they will parade themselves as a vet out there. And the farmers will not know, because farmers don’t care to ask these things.”* (KII, Government Veterinarian 3, November 2022)

Uncertified AHPs were considered by AHPs as unlikely to make farmers aware of ABWPs. In addition to the potential for AHPs to not make farmers aware of the ABWP, one government veterinarian described to us how AHPs may be motivated to administer antibiotics without clinical indication, meaning that farmers may not be aware that pigs had received antibiotics or of the ABWP associated with such ABU:

*“Some vets, some animal health technicians–along with a booster, they’ll give an antibiotic just to cover. So you don’t really know what that antibiotic is being injected for. It’s just so that they give more injections and get more money.”* (KII, Government Veterinarian 3, November 2022)

We also identified that actors other than AHPs could mislead farmers, meaning that farmers were unaware of the ABWP of administered antibiotics. During our study, participants often showed us substances that they administered to the pigs in order to improve productivity, such as multivitamin ‘boosters’, iron injections or other supplements. Although none of the products that participants showed to us and described to be used for such indications were labelled to contain antibiotics, it is possible that products may be incorrectly labelled meaning that farmers may not always be aware of any antibiotic content (see also [10, 39]). Highlighting a potential example of how this could take place, one participant showed us the ‘mycotoxin binder’ that they had purchased at a market which was a powdered substance that they reported to have been decanted into a clear plastic bag by the seller, although there was no labelling as to its composition; the participant subsequently added this substance to their pigs’ feed. Further, although we did not identify that any of the pig food kept on farms was labelled to contain antibiotics during the study, one AHP raised the possibility that antibiotics may be included in feed, unlabelled and therefore unknown to farmers (see also [39]).

*“So most of the animal feeds they won’t tell you, but they do put antibiotics*.” (FGD, Private AHP, November 2022)

It should be noted that other participants in the FGD vehemently disagreed with this participant’s statement.

Finally, whilst acquiring antibiotics for the ‘Drug Bag’, we identified that awareness of the specific ABWP of an antibiotic being administered could also be limited by the ABWP not being displayed on antibiotic packaging (see also [9, 10]).

*Theme 5*: *ABU practices challenge conformance with ABWPs*. Conversely to the ABU practices participants described as enabling ABWP conformance under Theme 2, other ABU practices may have acted as barriers to conformance with ABWPs. These were: a lack of recording of ABU meaning that ABWP could be misremembered; the possibility of ABU practices being different outside of our study period; and the potential for the extent of ABU to be under-reported by participants in our study.

The most common antibiotics which farmers reported had been used on their farms during our study had active ingredients including oxytetracycline (n = 10), trimethoprim sulphate (n = 9) and penicillin and streptomycin (n = 4). The antibiotics which farmers reported had been used most commonly (defined as those antibiotics which were reportedly used more than twice across all farms during the study period) had ABWPs displayed on packaging which were between five and 28 days. Participants in our study described how instances of ABU administered to pigs were rarely recorded by pig farmers (see also [22, 42]) and, as described in our related manuscript [28], the reasons for this appeared complex and were contextual. For example, both farmers and AHPs described how some AHPs hid the identity of antibiotics being administered to pigs, so that farmers could not purchase the antibiotic themselves in future without first seeking AHP assistance (see Scott et al. [28]). Without knowledge of the antibiotic, farmers described being unable to keep ABU records which might have included reference to the withdrawal periods. Further, as also described in our related work [28], farmers demonstrated errors in remembering time periods. Therefore, lengthy ABWPs of commonly used antibiotics represented a barrier to conformance with ABWPs in the context of rare medicine recording and recall problems.

For the possibility that ABU practices could be different outside of our study period, participants implied that ABU for near-slaughter-weight pigs could be higher during the cold season (from June to September), especially in pigs close to slaughter, meaning that conformance with ABWPs might be more regularly challenged over a different time than when our study took place. Here, participants discussed initiating antibiotic treatment for shaking pigs, which they described as having pneumonia.

“*Mainly, they get pneumonia when the weather changes. But when there is no change of weather, I don’t see any problem with them.”* (Interview, Farmer 11, October 2022)

For this purpose, one participant picked out of the ‘Drug Bag’ a gentamicin product which was labelled with a 45-day ABWP for kidneys that might be consumed. The ABWP for the consumption of meat (muscle tissue) after administration of gentamicin is just seven days, however offal (kidneys, liver, etc.) is commonly consumed in Kenya and is sometimes given as payment to slaughtermen at LIAs [16], meaning that antibiotics which concentrate in organs consumed as offal may hold specific risk.

We also experienced occasions where the lines between growth promotion and treatment of disease appeared blurry (see also [43]) meaning that ABU which constituted as being for growth promotion may have, in reality, occurred more frequently than farmers and AHPs reported in their more general discussions on the topic under Theme 2. For example, one participant reported the administration of antibiotics by an AHP to ‘*get them [the pigs] on proper weight’* and *‘bring back the body condition’* (Interview, Farmer 5 through translator, November 2022). Here, we were unable to ascertain whether pigs had been showing clinical signs of weight loss–possibly due to an infectious disease–or whether antibiotics were being used for growth promotion. In this way, it was not consistently possible to neatly divide instances of ABU into those for growth promotion, prophylaxis, metaphylaxis or treatment of disease to decipher whether antibiotics were used routinely in the production cycle. These gaps in our understanding were confounded by the methodological challenges we experienced determining ABU on farms, which we describe in our related manuscript [28] and included both over- and under-reporting of ABU.

*Theme 6*: *Economic fragility and resource constraints challenge ABWP conformance*. Under this theme, we identified that farmers’ social and economic circumstances did not consistently provide the opportunity for conformance with ABWP guidelines. In our study, financial precariousness and high production costs–mainly associated with increased feed prices–appeared to underline many farmer practices. These included whether farmers consulted AHPs, ABU practices and conformance with ABWPs. For the latter, by leaving farmers in a situation of financial fragility where they were not able to absorb costs around unforeseen events, farmer economic fragility appeared to prohibit delays in slaughter that might have been associated with ABWP conformance:

“Y*ou come in a situation whereby you are forced to do it [send a pig to slaughter within an ABWP]. You don’t have any other option. You don’t have rentals, you don’t have any other thing. And you have a situation so you sell the pig, you see? And it is not your fault. Even God can see, it is the situation that you are in.”* (Interview, Farmer 13, November 2022)

This was reflected in the only case of potential ABWP non-conformance identified during our study. As also described in our related manuscript [28], one farmer picked out nine different antibiotic-containing products from the ‘Drug Bag’ exercise completed on our final visit to the farm, as having been used in the last month. Although this account was not captured through any of the other methods we employed to determine farm-level ABU, the participant reported that these antibiotics had been administered by an AHP who had visited the farm in the second week of the study, with an aim to improve growth in near finishing-weight pigs. Notwithstanding the potential reliability issues with this reported instance of ABU (methodological challenges experienced in the current study are described in Scott et al. [28]), during the semi-structured interview, the participant described their intention to wait three weeks between the administration of medicines and slaughter of pigs. However, due to water scarcity on the farm (at the time of the study Kenya was suffering its worst drought for forty years; [44]), pigs were sold, on an emergency basis, just two weeks after the reported administration. This was within the ABWP for several of the antibiotics reportedly administered.

*“Ok so he [the farmer] is saying for most of these drugs, they were injected two weeks before. They were injected and then they were sold two weeks after.”* (Interview, Farmer 5 through translator, November 2022)

In this example, seven pigs which may have been within an ABWP were sent to slaughter at a LIA by this farmer. Across our small sample of 13 farms over a one-month study period, 14 pigs in total were reported to have been sent to slaughter at a LIA. (There was a total of 387 pigs on all 13 farms on the first visit.) In this way, for our study, resource scarcity may have represented a notable barrier to conformance with ABWPs for pigs sent to a LIA.

*Theme 7*: *Poor regulatory frameworks may weaken farmer motivation to conform with ABWPs*. In the final theme, AHPs described how the lack of enforcement around antibiotic residues at LIAs facilitated non-conformance with ABWPs.

*“You will find, if the pig is sent in a withdrawal period, it is taken to local slaughterhouses. It is really hard for it to be condemned. So the farmer will realise, ‘I sold my pig after a drug administration within say two weeks […] so why not repeat’*.” (FGD, Veterinary Para-professional, November 2022)

This view of poor regulation at the LIA amongst AHPs was described as being due to a lack of government funding to carry out enforcement activities such as meat inspection. Further, AHPs participating in our study reported instances of meat inspectors being undermined, bribed and pressured (see also [16]) to turn a blind eye to sick animals at slaughter and permit carcass damage suspected to be caused by injection of medication to be cut out of carcasses in order to avoid whole-carcass condemnation.

*“You [the farmer] had injected it yesterday, you don’t tell that person [meat inspector]. So during the slaughter, that site of injection is removed.”* (KII, Government Veterinarian 2, November 2022)

Participants described how this lack of enforcement was particularly utilised by brokers and traders, who were reported to buy pigs from farmers within ABWPs at a lower price but immediately send such animals for slaughter at a LIA.

Participant: *“Traders*, *I don’t think they ask [about ABWPs]*. *I don’t think so*. *Actually*, *the only reason they will ask is so that they buy it [the pig] at a lower price*. *Yeah*. *They will [say]*: *’Oh*, *this pig is sick*. *It has been treated for three days and*, *and it’s not getting better*.*’ It means the trader will buy it at a lower price*.*”*

Interviewer (CS): *“But sell it to [LIA] for the same price*?*”*

Participant: *“Yes*. *The only reason they will ask that is because of that [so that they can buy the pig at a lower price]*, *not because that they’re concerned about the withdrawal periods*.*”* (KII, Government Veterinarian 3, November 2022)

AHPs also raised the possibility that the perception of a tight regulatory and enforcement framework at the large integrated processor, relative to the LIA, might have led farmers to feel motivated to use LIAs as the salvage abattoirs for the county.

*“Also, if an animal falls sick just before slaughter and they [the farmers] are taking to [large integrated processor]–they had better take less animals. […] That is not the case if they’re going to the other local slaughterhouses. If that happens, the pig is taken for slaughter the same day, if they fall sick.”* (KII, Government Veterinarian 3, November 2022)

In such cases, whether or not ABWPs were conformed to would be dependent on whether antibiotic treatment had been completed before sending the pig to slaughter. For example, if a sick pig was sent straight to slaughter rather than being treated with an antibiotic, ABWPs would likely be conformed to. If antibiotic treatment was applied unsuccessfully and, instead, the LIA was used as salvage for non-recovering pigs, it is likely that ABWPs would not be conformed to.

## Discussion

By exploring the factors shaping ABWP conformance for farms supplying pigs to LIAs in Kiambu County, Kenya in a holistic manner, we have been able to describe enablers and barriers around conformance with ABWPs for this context.

We identified good awareness of ABWPs amongst the pig farmers we interviewed. Although there is not a large, well-tested body of literature on the topic of awareness around ABWPs, our findings that farmers were aware of ABWPs differed from several studies exploring this in East Africa [9, 37, 39]. For example, when surveying Maassai pastoralists in Tanzania, Mangesho et al. [45] reported that just 43.3% of their participants (n = 195) had heard of withdrawal periods. Showing different results, one study which surveyed livestock keepers in Kenya (none of which were reported to keep pigs) found that 89% of participants (n = 319) agreed with the statement that farmers should wait a period of time between administering antibiotics to animals and consuming or selling milk or animal products [46]. The relatively high level of awareness experienced in our study may have been influenced by factors such as the peri-urban location of our study which could affect, for example, the number of AHPs that study and therefore remain practising in proximity to the city of Nairobi [20]. Similarly to our study, in their PhD thesis, Njoroge [25] reported that 95% of surveyed cattle smallholders in Kiambu county (n = 99) had some knowledge of medicine withdrawal periods. Furthermore, the presence of the large integrated processor for pigs may also have affected participating farmers’ awareness, potentially leading to greater knowledge transfer through their extension workers and spill-over to the domestic market.

Our result that five out of the 13 participating farmers could not explain the meaning of an ABWP from its indication on antibiotic packaging could be reflective of what was found by Ndukui et al. [47], where poultry farmers described language on medicine inserts to be challenging. Together, our results and those of Ndukui et al. [47] indicate value in simplifying the language around ABWPs on antibiotic packaging. Given that three out of the five farmers who could not identify the ABWP from an item of antibiotic packaging did not speak English (see Table 1), it may also be valuable to translate ABWP information into the national languages for the particular country within which a medicine is being sold. Suspicions that products may be incorrectly labelled meaning that farmers may not always be aware of any antibiotic content have also been reflected in results gathered by other researchers in Malawi [39] and Kenya [10]. These findings indicate a need to ensure that antibiotic inclusion and associated ABWPs are reliably and clearly displayed.

Whilst several farmers reported that AHPs made them aware of ABWP, AHPs themselves reported potential variability in whether they (AHPs themselves) make farmers aware of ABWPs at each administration. This reflects findings from other research in East Africa; conversations around ABWPs were not observed to take place between AHPs and farmers who kept free-roaming farm animals or housed farm animals on a small scale in Malawi [39]. The tensions we identified between veterinary para-professionals and veterinarians are reflective of findings by Arvidsson et al. in Uganda [41], where veterinarians characterised para-veterinary professionals as offering poor advice to farmers which could contribute to the spread of disease while veterinary para-professionals characterised themselves as possessing greater local knowledge than veterinarians. Further, variable AHP practices reported in our study, such as not informing farmers of ABWPs or AHPs hiding the identity of medicines, introduces the idea that ABU under AHP supervision does not necessarily equate to prudent practices, as has also been discussed for poultry layer farmers in Ghana and Kenya [14].

Concerns that AHP behaviours could contribute to non-conformance with ABWPs could, however, be challenging to influence. Firstly, uncertified AHPs could be very difficult to reach for knowledge exchange interventions. Secondly, whether or not an AHP explains an ABWP to a farmer upon prescribing a particular antibiotic may depend on several factors. This could include the age of the pig undergoing treatment and the perceived relevance of this information, as well as the context in which AHPs are prescribing medicines. Therefore, any interventions aimed at improving AHP-farmer conversations around ABWPs at the point of prescription or administration of antibiotics would need to account for the factors influencing these discussions as well as the context in which antibiotics are prescribed.

Reflected in results from other studies in Kenya [22, 42], we also identified a lack of medicine recording. This was in the context of lengthy ABWPs of commonly used antibiotics (the antibiotics reportedly used in our study were similar to the types of antibiotics described in a pilot study for pigs in the same county [21] and by Rware et al. [46] for livestock in Kenya) as well as time recall problems. A lack of recording of ABU may, therefore, represent a notable barrier to ABWP conformance and could mean that improving conversations around ABWPs at the point of antibiotic prescribing or administration may not result in improved conformance with ABWPs.

Our finding that all farmers in the current study described an intention to conform with ABWPs is broadly reflective of work by Ndukui et al. who found that 82% of poultry farmers in the same county considered it important to conform with ABWPs [47]. In our study, farmers described how their caring intentions provided them with strong motivations to conform with ABWPs. That being said, the discrepancies in findings between farmers’ intentions to comply with ABWPs as reported under Theme 2 and AHP perceptions of farmers’ intentions (who believed that lack of poor regulatory frameworks motivated farmers not to comply) were interesting to explore and warrant further research. If farmer intentions experienced in this study were found to be representative of farmers in the area, the discrepancies we experienced raise potential challenges for researchers interviewing a small number of AHPs as a proxy to understand knowledge, attitudes and practices amongst a larger group of farmers, as these results may not consistently triangulate between groups.

Although previous studies have indicated a potential value in introducing regulation around conformance with ABWPs for this context [18], our findings and the work of others indicate that the introduction of effective regulation would be problematic. ABWPs are not currently part of Kenyan law for pigs not intended for export and LIA infrastructure was such that pork was not tested for antibiotic residues at the time of the study by Bor et al. [18]. Complicated chains of sellers have been described as providing challenge to farm-to-processing traceability [19] and our findings suggest that the marginalisation of farmers may leave them vulnerable to being misled and unfairly blamed. We also identified that corruption and underfunding of county government veterinary officials could challenge policing and enforcement of further legislation. As has been raised by Murungi et al. [16], additional regulation around ABWPs could inadvertently act to encourage food safety hazards such as slaughter of sick animals or home slaughter for human consumption.

During the study, we frequently interpreted economic fragility to be underlining both farmer and AHP behaviours which, in turn, could lead to both enablers of and barriers to conformance with ABWPs. These included the avoidance and minimisation of ABU in order to avoid associated financial costs. Our finding that ABU for growth promotion was rare in our study context is reflective of findings by Irungu et al. [21] during a pilot study in neighbouring counties including Kiambu, as well as some authors’ findings across Kenya [48, 49] (although see [46, 50] for the converse in poultry). Our findings of very targeted ABU in pigs, however, were different from results found by Bor et al. [18] who, whilst sampling pork meat from a LIA in the county we studied, found that pig lesion score was not significantly associated with the presence of antibiotic residues. Our finding that younger pigs were the most likely to receive antibiotic treatment was also not reflected by the analyses completed by Bor et al. [18] as they found pig live weight not to be significantly associated with positivity for antibiotic residues, though the mean pig live weight was slightly lower for the group that was positive for antibiotic residues. Both these factors in the analyses by Bor et al. [18] suggest more indiscriminate or different targeting of ABU within their study context than our farmer participants indicated.

Also reflective of the importance of economic fragility as a driver of practices in our study, social and economic context appeared to be pivotal determinants of whether farmers could absorb the economic losses associated with conformance with ABWPs during times of adversity, such as in the face of resource scarcity on the farm. This is reflective of other studies of cattle and poultry in Kenya, where researchers have described farmers’ financial situation and economic concerns to necessitate animals being sent to slaughter within ABWPs, or animal produce to be used [14, 22, 50].

## Conclusions

This study provides an important foundation for those seeking to positively influence conformance with ABWPs and for future research which examines the enablers and barriers which we identified in more detail. We highlight the complex and intersectional nature of the relationships that drive ABU and engagement with ABWPs, with which future work and successful interventions must actively engage.

In addition to providing important insights for those seeking to improve conformance with ABWPs in this context, our study also contributes to the global body of research examining ABU practices on farms. Our findings support the growing body of evidence acknowledging that knowledge alone may not be enough to promote behavioural change around ABU [45, 46, 51–54]. Our results add weight to the notion that, if interventions are to be successful, we must consider and account for both the complexity of the system and the importance of contextual factors as drivers of practice [50, 54, 55].

## Limitations

Given that disease profiles can be seasonal [56], it is reasonable to expect that ABU may also be seasonally influenced [57]. Therefore, the results of this study may be considered appropriate for the short rain period in Kenya; further work would be required to capture seasonal variations. We recruited a small number of farms from just four sub-counties and our methods of recruitment (which involved the assistance of government veterinarians) may have introduced selection bias. Whilst the comprehensive exploration of interactions between demographic characteristics and ABWP conformance enablers and barriers would make a fertile topic for future research, these dynamics were outside of the scope of this study. Finally, the positionality of researchers completing this study (see positionality statement above) will have affected the lens through which the research was completed, the data that were collected and the analytical results. Therefore, our findings may not be generalisable to all pig farmers in Kiambu County supplying a LIA and different results may have been obtained by other researchers exploring this topic.

## Supporting information

S1 Checklist Checklist. Inclusivity in global research checklist containing additional information regarding the ethical, cultural and scientific considerations specific to inclusivity in global research for our study exploring conformance with antibiotic withdrawal periods in Kiambu County.
(DOCX)

S1 Fig Feedback booklet. The feedback booklet which was made by researchers based on our observations during our study exploring conformance with antibiotic withdrawal periods in Kiambu County. This booklet was translated into Swahili and given to farmers at the end of the study.
(PDF)

S1 File Interview guides. Farmer and animal health professional interview and focus group discussion guides for our study exploring conformance with antibiotic withdrawal periods in Kiambu County.
(PDF)

S2 File Medicine recording materials. The medicine recording sheet (with English translation) that was left with farmers to record instances of medicine use during the study as well as a photo of the waste bucket, clipboard and associated signage which was also left with farmers.
(DOCX)

## Abbreviations

ABU: antibiotic use
ABWP: antibiotic withdrawal period
AHP: animal health professional
FGD: focus group discussion
HP-CIA: highest priority critically important antimicrobial
KAP: knowledge attitude and practices
KII: key informant interview
LIA: local independent abattoir
LMIC: low-and middle-income country
SSI: semi-structured interview
