# Supplementary material for: A qualitative exploration of the enablers of and barriers to conformance with antibiotic withdrawal periods on smallholding, peri-urban pig farms in Kiambu County, Kenya
Source: PLoS One. 2025 Jan 10;20(1):e0312362. doi: 10.1371/journal.pone.0312362 (PMC11723525; doi:10.1371/journal.pone.0312362)
Supplement: S1 Fig — The feedback booklet which was made by researchers based on our observations during our study exploring conformance with antibiotic withdrawal periods in Kiambu County. This booklet was translated into Swahili and given to farmers at the end of the study. (PDF) [file pone.0312362.s002.pdf]

Feedback for small-scale pig farmers in Kiambu County

Biosecurity and use of medicines:

Biosecurity with pigs

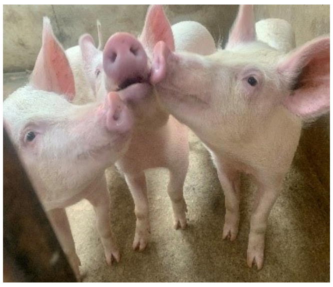

New pigs

3 metres separation

Old pigs

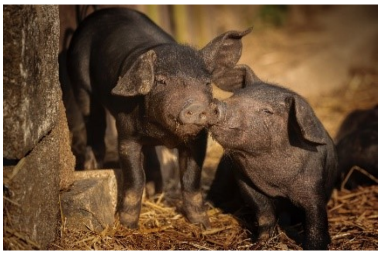

Buy new pigs from trusted farms without disease. When you buy in new pigs, keep them separate from your own pigs for two weeks while you monitor them and check they are not sick.

Biosecurity with people

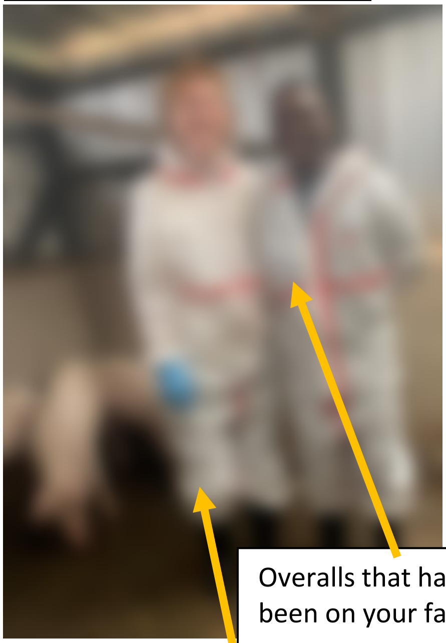

Overalls that have only been on your farm

Your old pair of gum boots

Before anyone comes on the farm, get them to put on gum boots and overalls from your farm so they don't bring on diseases.

Cleaning and disinfectant

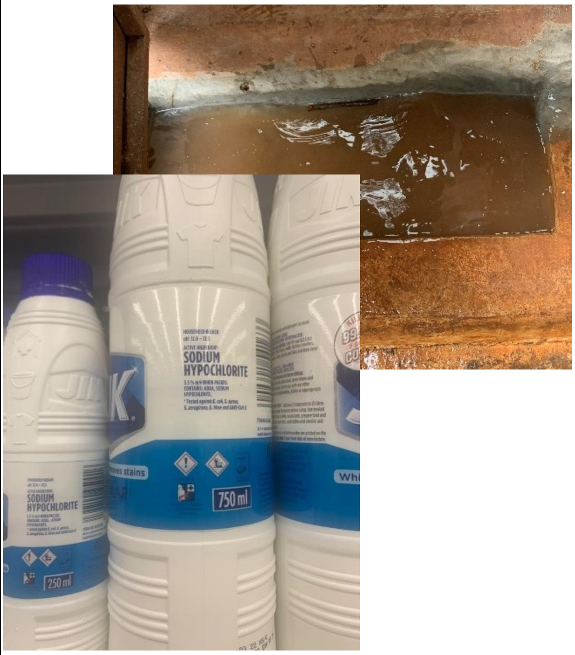

If you are using JIK for disinfectant, use one part JIK to six parts water. To kill African Swine Fever, equipment and boots must sit in JIK for 15 minutes.

Using medicines

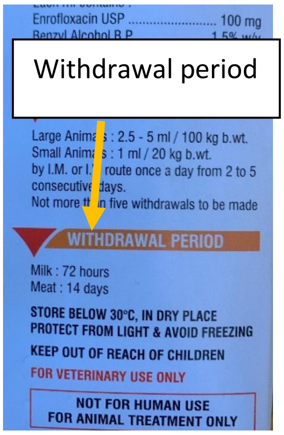

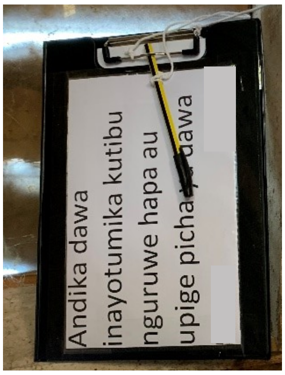

Only use medicines as instructed by your vet. Keep a record of any pig sickness and any medicines used. After giving a pig a medicine, wait the withdrawal time before you send it to slaughter. The withdrawal time will be shown on the back of the medicine and is the time you must wait between giving the medicine and sending the pig to slaughter.

Water and feeding:

Water access

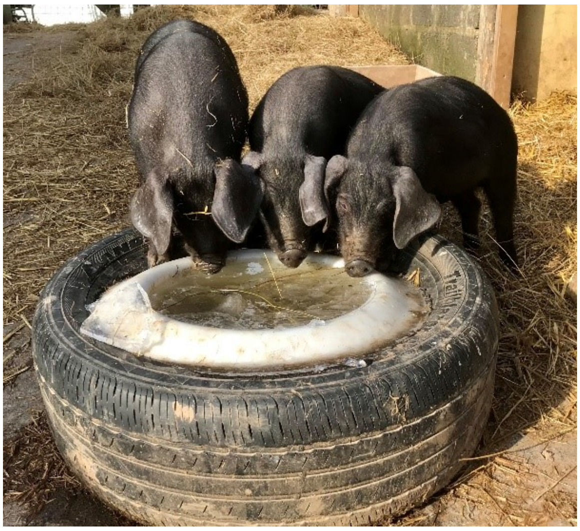

Pigs need constant access to clean, fresh water. This is even more important if they are breast-feeding as producing milk for strong piglets requires a lot of water. Breast-feeding sows will drink up to 50 litres of water per day.

Feeding pigs

From behind, pigs should look like this:

Not this:

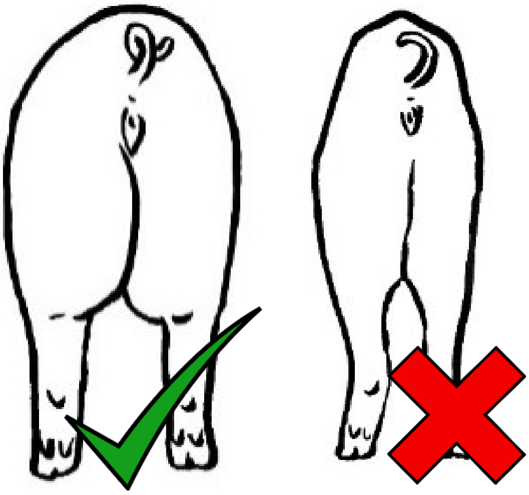

Give pigs enough food so that you can’t see their ribs, hip bones, shoulders or backbone. You should only be able to feel these bones when pressing with your fingers.

Feeding sows

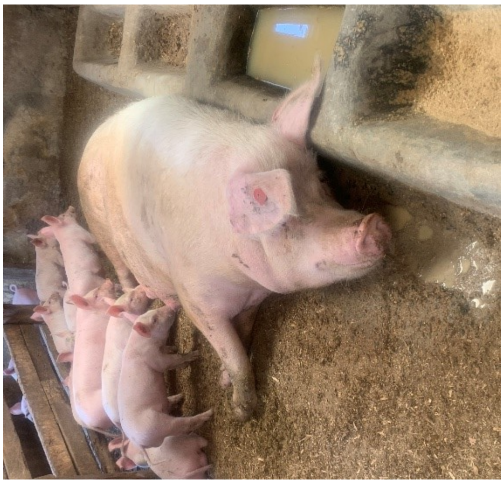

Feed sows as much as possible whilst breastfeeding, several times per day. Only feed good quality pig food at this time. Then you will be able to wean stronger piglets and mate the sow again 5 days after weaning without needing a break for her to gain weight. Milk production peaks when piglets are around three weeks old, so the sow will need the most food at this time.

Hotel or kitchen waste

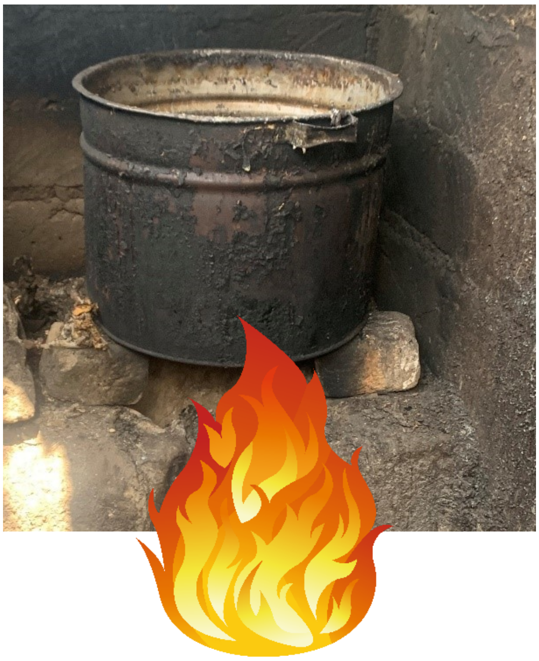

Boil kitchen or hotel waste before you give it to the pigs to kill diseases like African Swine Fever.

Amount of pig food to feed:

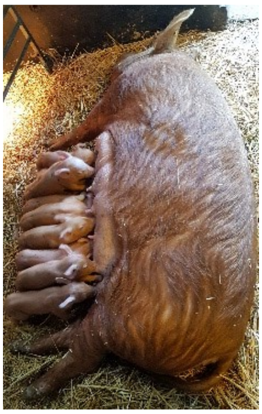

<u>Sow breast-feeding:</u>

Increase by 0.5kg per day to as much as she will eat.

This should be over 10kg per day at three weeks after birth.

<u>Weaned sow:</u>

Gradually decrease feed back down to 2-2.5kg per day.

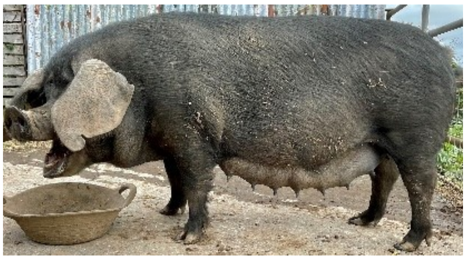

<u>1 month old piglet:</u>

0.5kg per day

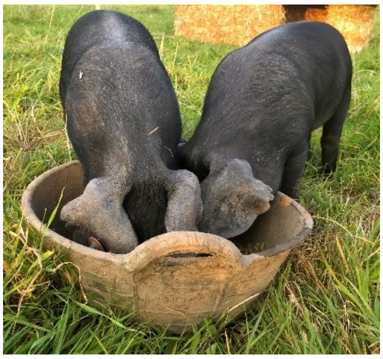

<u>2 month old piglet:</u>

1kg per day

<u>3 month old piglet:</u>

1.5kg per day

<u>4 month old piglet:</u>

2kg per day

<u>5 month old pig to slaughter:</u>

2.5-3kg per day

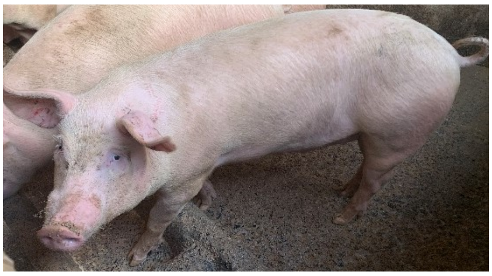

<u>Pigs over four months old separated for breeding</u>:

2-2.5kg per day. Increase by 0.5kg for the week before and the week of mating for both sows and boars to increase the number of piglets born.

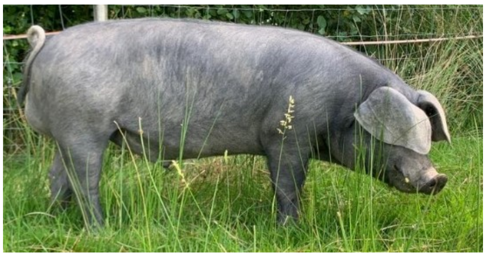

<u>Last month of pregnancy:</u>

2.5-3kg per day

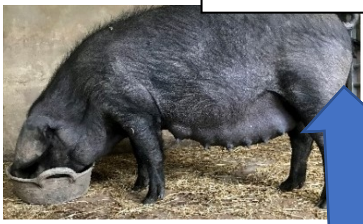

* Approximate weights of good quality commercial pig feed. Supplementary food will be nutritionally different.

Breeding and rearing piglets:

Signs of heat

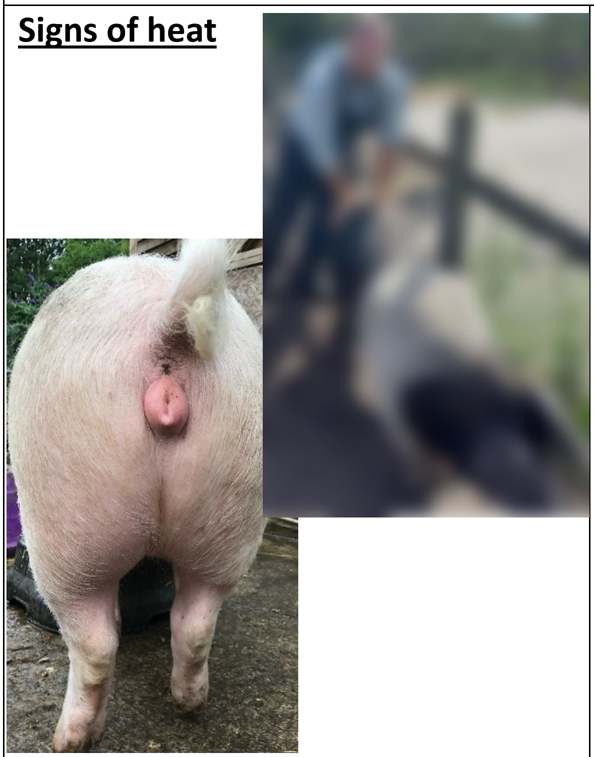

Signs of heat are a red and swollen vulva, interest in boars and standing heat. Standing heat means that she will stand rigidly if you press on her bottom. This happens every 21 days. It will normally happen around five days after weaning piglets.

When to serve

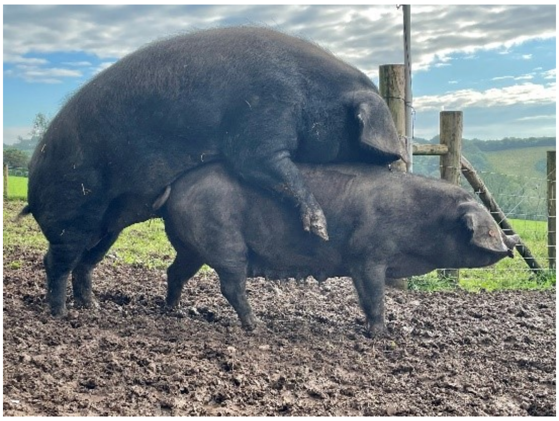

Do not serve a female pig on her first heat, wait until she is over 140kg and 7 months old. Serve 12 hours after she starts standing heat and again 12 hours later. Before serving, make sure that she has at least 14 productive teats to be able to breast-feed lots of piglets. If the service was successful, she won't come back on heat 21 days later.

A piglet area

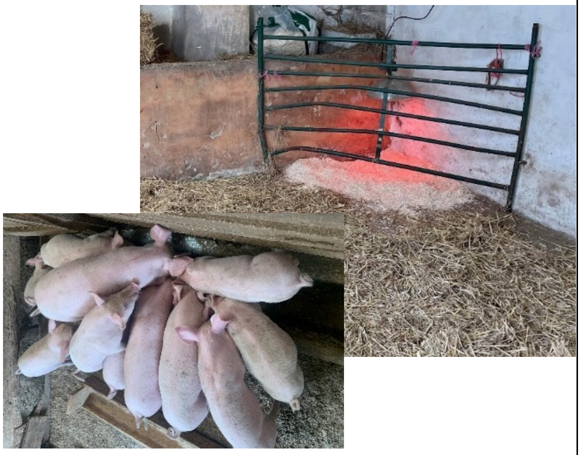

Give piglets a separate creep area with fresh bedding so that they don't lie near the sow for warmth. Then they will be less likely to get laid on by her and killed. If you cannot make a creep area, provide piglets with some bedding in a corner. From two weeks of age, feed piglets creep feed separately from the sow to ease their transition at weaning.

Docking tails

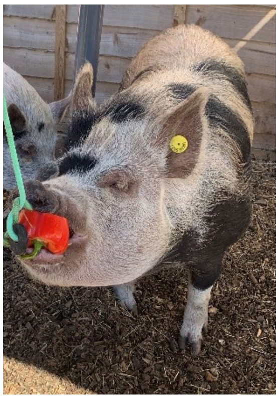

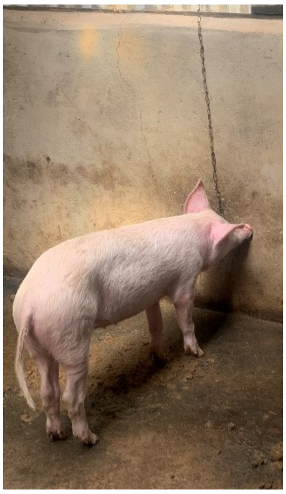

Don't dock tails unless you have a problem with pigs biting each other's tails. Docking tails does not make pigs grow faster or breed more easily. Piglet leg problems can be caused by docking tails or clipping teeth. If you have a problem with pigs biting tails or ears, hang some chains or pig safe toys for them to play with and avoid boredom.
