# Supplementary material for: A qualitative exploration of the enablers of and barriers to conformance with antibiotic withdrawal periods on smallholding, peri-urban pig farms in Kiambu County, Kenya
Source: PLoS One. 2025 Jan 10;20(1):e0312362. doi: 10.1371/journal.pone.0312362 (PMC11723525; doi:10.1371/journal.pone.0312362)
Supplement: S2 File — The medicine recording sheet (with English translation) that was left with farmers to record instances of medicine use during the study as well as a photo of the waste bucket, clipboard and associated signage which was also left with farmers. (DOCX) [file pone.0312362.s004.docx]

**CHATI YA KUREKODI DAWA (Medication recording chart)**

| **Tarehe**  (Date) | **Jina la dawa lililotumiwa?**  (Name of the medicine) | **Sababu ya kutumia hii dawa – ugonjwa gani?**  (Reason for using the medicine?) | **Iliyotolewa na nani?**  (Administered by whom?) | **Kwa kikundi gani ya nguruwe?**  (Administered to which pigs?) | **Matokeo ya kutumia dawa hii?**  **(Nguruwe walipona,  Nguruwe walikufa, Nguruwe waliuzwa, Hakuna mabadiliko?)**    (The outcome of administering the medicine - pigs improved? Pigs died? Pigs sold?) |
| --- | --- | --- | --- | --- | --- |
|  |  |  |  |  |  |
|  |  |  |  |  |  |
|  |  |  |  |  |  |
|  |  |  |  |  |  |


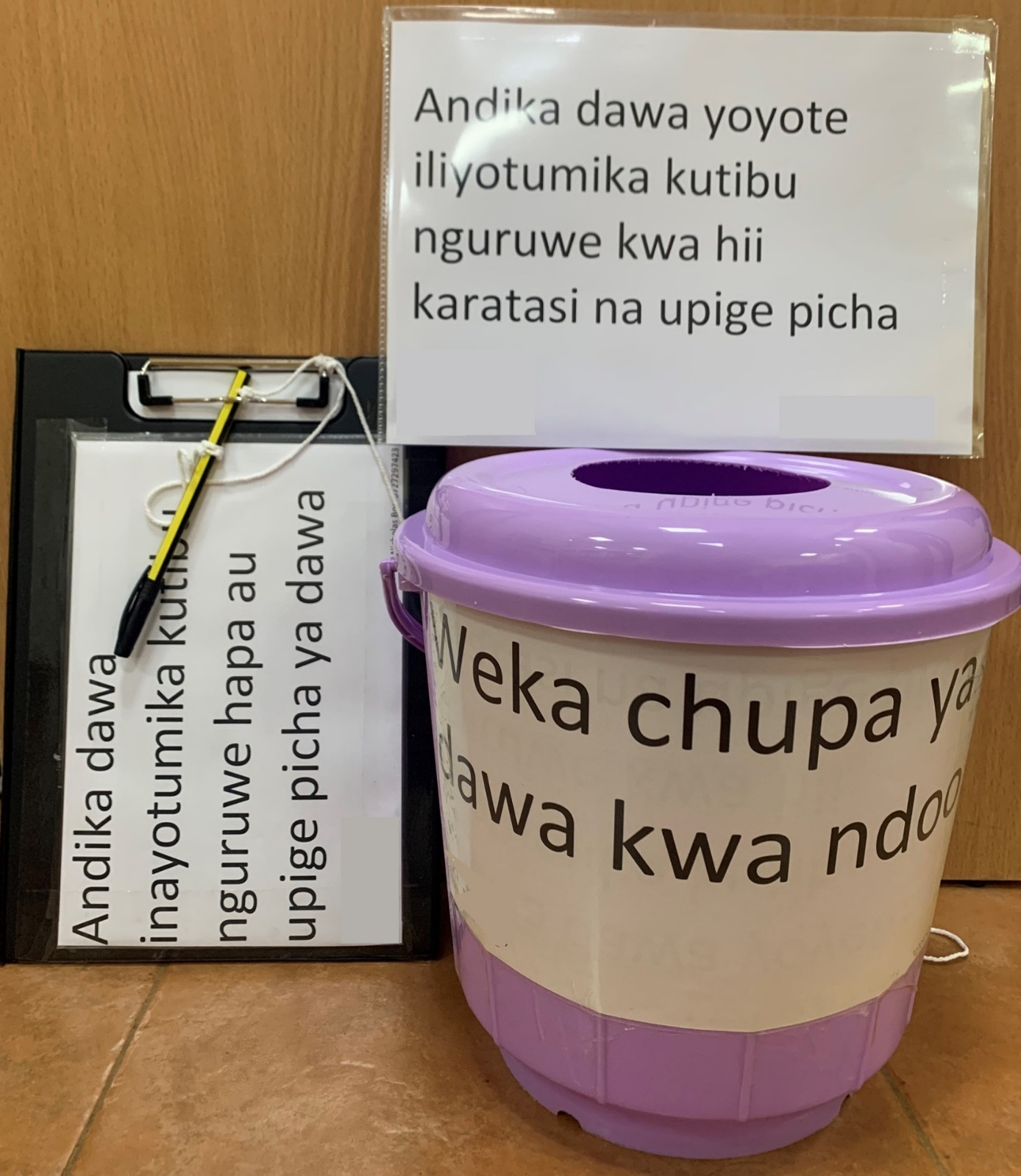


Write any medicine used to treat the pigs on this paper and take a picture.

Put the medicine bottle in the bucket.

Write the medicine used to treat the pigs on this paper or take a picture of the medicine.
